# Association of Screen Time Exposure With Autism Spectrum Disorder in Four to Six-Year-Old Children in Arar City, Saudi Arabia

**DOI:** 10.7759/cureus.61447

**Published:** 2024-05-31

**Authors:** Shehab A Alenazi, Sawsan H Hasham, Irfan Hanif, Sarwar Hussain, Mohammedalamin Abderahim, Abdulrahman M Alanazi, Bandar F Alhudhayyiri, Abdullah F Alanazi, Abdulrahman M Alanazi, Ekramy Elmorsy

**Affiliations:** 1 Pediatrics, Northern Border University, Arar, SAU; 2 Psychiatry, Erada and Mental Health Complex, Arar, SAU; 3 Medical School, Faculty of Medicine, Northern Border University, Arar, SAU; 4 Pathology, Northern Border University, Arar, SAU

**Keywords:** child’s screen time, arar city, arar, screen time, screen exposure time, autism spectrum disorder

## Abstract

Background

Autism spectrum disorder (ASD) is a psychopathologic disorder caused by several factors. The early signs include poor interaction and communication, delayed milestones, and repeated behavior patterns. This study aimed to assess the relationship between screen time and ASD severity and investigate the types of electronic devices associated with ASD in children aged four to six years in Arar City, Kingdom of Saudi Arabia (KSA).

Methodology

A cross-sectional study was conducted in primary healthcare centers (PHCs) in Arar City, KSA. The study enrolled all parents with children aged four to six years attending the PHCs in Arar City, KSA.

Results

The total sample size was 199 participants. Regarding the relationship between screen time exposure and ASD, there were variable screen time exposure durations, with 22.6% of children exposed for less than an hour, 30.7% for one to two hours, and 46.7% for more than two hours. Moreover, the type of electronic devices to which children were exposed varied, with smartphones being the most prevalent (68.3%). In terms of the age of children since exposure to electronic devices, the data indicated that 30.2% were exposed before the age of two, 35.2% between two and three years, and 34.7% after three years of age. Regarding the relationship with sociodemographic characteristics, there was a statistically significant relationship with the mother’s age at birth (p = 0.050), mother’s education level (p = 0.009), father’s education level (p = 0.049), whether the child was suffering from any chronic or neurological disease (p = 0.008), age since the child was exposed to electronic devices (p = 0.049), and screen time exposure duration (p = 0.040).

Conclusions

The study highlights the significant association between screen time exposure and the development of ASD in children. Public awareness of this associated risk among caregivers is recommended to follow the protective guidelines. Further research and interventions are needed to better understand and address the impact of screen media use on children’s neurodevelopment and overall well-being.

## Introduction

Autism spectrum disorder (ASD) is a psychopathologic disorder caused by various biological, psychological, environmental, and genetic factors. The environmental factors, including prenatal and postnatal factors, lead to a 50% risk of development of ASD [1]. Early signs of ASD include difficulty interacting with others, poor communication skills, and repeated behavior patterns [2].

The use of electronic screen media prevails more in children under three years of age, and there are chances of an increase in its prevalence in the next decade [3]. In 2000, ASD prevalence was 6.7/1,000 in eight-year-old American children. While in 2010, this prevalence increased to 14.7/1,000, and, in 2014, it reached 16.8/1,000 [4]. Globally, kids who were more socially active in the past are now more involved in screen use. However, screen use at an early age causes alterations in the brain, either neurochemical or anatomical, as screen-addicted kids were shown to have limited melatonin concentration in their brains [5].

Several studies have demonstrated the association between increased screen use and adverse effects on health [6-9]. These effects include reduced cognitive capability, poor social interaction and language development, mood disorders, and autism-like behaviors, such as hyperactivity, irritability, limited attention span, and altered capability to process the social and psychological information that needs time and reflection to absorb [6-8]. These effects lie under ASD. In addition, some theories consider screen use a potential factor of ASD. Heffler and Oestreicher [9] showed that infants’ exposure to audiovisual input causes disturbance in the brain pathways. It causes the brain’s non-social processing of audio and visual stimuli and ultimately affects social skills and cognitive development. These alterations in brain activity cause ASD in kids.

The American Academy of Pediatrics recommends the criteria for age-specific use of screen media to maintain the advantages and disadvantages. Children aged two to five years are not recommended to watch the screen for more than one hour per day. Children older than five years should watch good TV programs with restrictions to avoid the negative impact of screen media on children’s behavior, sleep patterns, and other health issues [10]. The World Health Organization (WHO) recommends a watch time of one hour a day for children aged four to six years [11]. Additionally, screen use of more than the recommended time at the ages of four to six years was shown to alter the children’s developmental milestones within the domains of socio-emotional abilities, language or communication abilities, and cognitive abilities [12-16]. At the brain level, Hermawati et al. showed that excessive screen use reduced the activity of melanopsin-expressing neurons and gamma-aminobutyric acid neurotransmitters with declined cognitive and language development [17].

The Social Communication Questionnaire (SCQ) was developed as a screening tool and is based on the revised version of the Autism Diagnostic Interview (ADI) [18]. It is not a diagnostic tool and children identified as at risk of ASD from the SCQ warrant an autism-specific diagnostic evaluation. However, it is widely used as a screening tool to assess the association between screen exposure time and the development of ASD [19,20]. Hence, this study was conducted to study the relationship between ASD and electronic devices and the relationship between screen time and ASD severity among Saudi children aged four to six years using SCQ via interviewing caregivers attending primary healthcare centers (PHCs) in Arar City, Kingdom of Saudi Arabia (KSA).

## Materials and methods

This cross-sectional study was conducted from December 2023 to March 2024. Out of 11 PHCs in Arar City, three PHCs with the highest patient attendance rates were chosen for this study for a better representative sample from three districts of Arar. Each center contributed to 70 parents with children aged four to six years who were selected randomly. Three visits were done per week for each center. During each visit, 10 eligible mothers were selected randomly by systematic random sampling technique. Enrolled participants were Saudi mothers with children aged four to six years attending PHCs in Arar City, KSA who were willing to provide informed consent after reading and understanding the research background and aims. Parents with children having severe physical, neurologic, and chronic disorders were not enrolled in the study. All participants’ data were kept confidential through all phases of the research project.

Sample size

The sample size was calculated based on the sample size formula as follows: n= [Zα/d]2×p [1-p] [21], where n is the sample size; zα (for α = 0.05) = 1.96; d is how close to the proportion of interest, the estimate is desired to be, which is assumed 0.05; P is the expected proportion according to a previous study conducted by Alrahili et al. in Saudi Arabia that reported high SCQ scores among 14.3% of the studied participants [19]. The minimum sample size calculated for the study was 189 parents.

Data collection instrument

The questionnaire was prepared as an online form as well as a face-to-face interview using an Arabic questionnaire. The validation of the Arabic translation of the questionnaire was checked among 20 participants and the results were checked to ensure proper translation and understanding of the questions by the participants. Validation participants’ responses were not included in the final analyzed data. The questionnaire comprised three main parts. The first part covered sociodemographic data (parental age, marital status, educational level, income, occupation, nationality, consanguinity with the husband,) and child characteristics (age, gender, birth order). The second part included questions about screen exposure (television, mobile, tablet/iPad, computer, others), the age of starting exposure to screens, and daily hours spent in front of it. The third part was the Arabic version of SCQ, which was previously used and validated by Aldosari et al. (2019) to assess the child’s social interaction and behavior [22]. The SCQ consists of 40 items used to screen for symptoms associated with ASD. The 40 items are dichotomous, with “Yes” or “No” answers. Item 1 is only used to assess whether the child can speak with short phrases or sentences, while items 2 through 40 are used for the actual scoring. Thus, the total score ranges from 0 to 39. A score of 1 is given for the presence of abnormal behavior and a score of 0 for its absence. Items 2, 9, and 19 through 40 are negatively worded, where 1 is given for the response option “No” and 0 for “Yes.” For items 3 to 8 and 10 to 18, a score of 1 is given if the answer is “Yes” and 0 otherwise.

Data analysis

Data analysis was done using SPSS software version 20 (IBM Corp., Armonk, NY, USA). Categorical variables were summarized as counts and percentages. ASD was considered at SCQ score ≥15. Comparisons between groups were made using appropriate statistical statistics. A multivariate logistic regression model was used to study the effect of different variables on the outcomes. A p-value >0.05 was considered statistically significant.

Administrative and ethical considerations

The study design was approved by the Local Bioethics Committee of Northern Border University (approval number: 107/23/H). Subsequently, it was submitted to Northern Borders General Health Affairs. The approval was also obtained from the administration of PHCs of Northern Borders General Health Affairs. A permission letter was sent to Western Psychological Services Publishing Company (rights@wpspublish.com) to use the validated Arabic version of SCQ.

## Results

The various demographic parameters of the enrolled participants are shown in Table 1. The distribution of mothers’ ages at birth indicates a varied range, with a notable proportion (72.9%) falling within the 20 and 34-year age groups. Regarding maternal occupation, a significant percentage were identified as housewives (50.8%), while a substantial proportion (44.2%) were employed in the government sector. Notably, all participants in the study were of Saudi nationality, highlighting a homogenous demographic in this regard. The education levels of mothers varied, with a majority (66.8%) holding a college degree or higher. Most mothers (92.5%) were reported as being married, with a smaller percentage being divorced or widowed. The data also shed light on the degree of kinship among participants, with a slight majority (58.3%) indicating a degree of kinship. Family income distribution showed a significant proportion (55.8%) earning more than 10,000 SAR monthly. The number of family members varied, with a substantial percentage (45.2%) having more than five members. Child-related data revealed a mix of ages and genders among the participants, with a notable proportion (73.9%) of male children. Additionally, a significant percentage (19.1%) of children were born preterm, with varying birth orders observed.

**Table 1:** Sociodemographic characteristics of participants. Data are presented as numbers and percentages (n = 199).

Parameter		No.	Percent (%)
Mother’s age at birth	Less than 20	22	11.1
	20 to 34	145	72.9
	35 or above	32	16.1
Mother’s occupation	Government employee	88	44.2
	Private sector	7	3.5
	Housewife	101	50.8
	Others	3	1.5
Nationality	Saudi	199	100.0
	Non-Saudi	0	0
Mother’s education level	Primary school	13	6.5
	Middle school	13	6.5
	High school	25	12.6
	College degree or higher	133	66.8
	Uneducated	15	7.5
Father’s occupation	Government employee	156	78.4
	Private sector employee	18	9.0
	Retired	1	0.5
	Freelancer	8	4.0
	Unemployed	16	8.0
Father’s education level	Primary school	6	3.0
	Middle school	15	7.5
	High school	40	20.1
	College degree or higher	127	63.8
	Uneducated	11	5.5
Mother’s marital status	Married	184	92.5
	Divorced	10	5.0
	Widowed	5	2.5
Degree of consanguinity	No	83	41.7
	Yes	116	58.3
Family monthly income in SAR	Less than 5,000	12	6.0
	5,000 to 10,000	76	38.2
	More than 10,000	111	55.8
Family members	Less than 4	53	26.6
	4 to 5	56	28.1
	More than 5	90	45.2
Child’s age	4	78	39.2
	5	37	18.6
	6	84	42.2
Child’s gender	Female	52	26.1
	Male	147	73.9
Child’s birth was preterm	No	161	80.9
	Yes	38	19.1
Child’s birth order	First	59	29.6
	Second	34	17.1
	Third	27	13.6
	Fourth	79	39.7

As illustrated in Table 2, the data presented provides valuable insights into children’s screen time exposure and its associated parameters. Among the 199 children studied, a significant majority (85.9%) did not suffer from any chronic, neurological, or debilitating disease, while 14.1% reported such conditions. The type of electronic devices to which children were exposed varied, with smartphones being the most prevalent (68.3%), followed by iPads (24.1%), computers (5.0%), televisions (2.0%), and PlayStations (0.5%). In terms of the age of children since exposure to electronic devices, the data indicated that 30.2% were exposed before the age of two, 35.2% between two and three years, and 34.7% after three years of age. Furthermore, the screen time exposure duration varied, with 22.6% of children exposed for less than an hour, 30.7% for one to two hours, and 46.7% for more than two hours.

**Table 2:** Parameters related to childrens’ screen time exposure. Data are presented as numbers and percentages (n = 199).

Parameter		N	Percent (%)
Does the child suffer from any chronic, neurological, or debilitating disease?	No	171	85.9
	Yes	28	14.1
Type of electronic device the child is exposed to	Computer	10	5.0
	iPad	48	24.1
	PlayStation	1	0.5
	Smartphone	136	68.3
	Television	4	2.0
Age of child since exposed to electronic devices	Less than 2 years	60	30.2
	2 to 3 years	70	35.2
	More than 3 years	69	34.7
Screen time exposure duration	Less than an hour	45	22.6
	From 1 to 2 hours	61	30.7
	More than 2 hours	93	46.7

The effect of the various parameters considered in the study on the children’s behavior and communication skills with a sample size of 199 participants is shown in Table 3. Each parameter is divided into “Yes” and “No” responses, shedding light on different aspects of the children’s behavior. For instance, the data showed that a significant percentage of children (74.9%) could talk using short phrases or sentences, while a smaller percentage (25.1%) could not. Additionally, the research delved into more specific behaviors such as the child’s ability to engage in conversations involving role-playing or basing their words on previous statements, their use of socially inappropriate questions or statements, confusion with pronouns, repetitive behaviors, and unusual interests, among others.

**Table 3:** Participants’ knowledge of the association of screen time exposure with autism spectrum disorder in children. Data are presented as numbers and percentages (n = 199).

Parameter	Responses	Yes	No
Can the child speak using short phrases or sentences? If no skip to question number 8	n	149	50
	%	74.9%	25.1%
Can you have a conversation with the child that includes exchanging roles or basing his/her words on what you said? (n = 179)	n	134	45
	%	74.86%	25.14%
Has the child ever used strange expressions or said the same words repeatedly in the same way (whether phrases he/she heard from other people or ones he/she says himself)? (n = 177)	n	97	80
	%	54.8%	45.2%
Has the child ever used socially inappropriate questions and statements? For example, does he/she have a habit of asking questions or making personal comments at awkward times? (n = 180)	n	66	114
	%	36.67%	63.33%
Has the child ever confused the pronouns? For example, to say “you” or “he” or “she” instead of “I”? (n = 179)	n	75	104
	%	41.9%	58.1%
Has the child ever used words that sound like he/she invented or made them up himself/herself, or formulated phrases in a strange or indirect way, or did he/she use his/her own way to express specific things (such as saying, “hot rain” to express “steam”?) (n = 180)	n	81	99
	%	45%	55%
Has the child ever said the same thing repeatedly in the same way, or did he/she insist that you say the same thing over and over again? (n = 177)	n	78	99
	%	44.1%	55.9%
Has the child ever done things that he/she seems forced to do in a certain way or order or any ritual that he/she insists that you do?	n	81	118
	%	40.7%	59.3%
Did the child’s facial expressions ever seem appropriate to the situations, to your knowledge?	n	134	65
	%	67.3%	32.7%
Has the child ever used your hand as a tool or as an extension of his arm, such as pointing with your finger or placing your hand on the doorknob to open the door?	n	89	110
	%	44.7%	55.3%
Has the child ever had any unusual interests that might seem unusual to other people (for example, traffic lights, drainpipes, or timetables?)	n	79	120
	%	39.7%	60.3%
Has the child ever seemed more interested in a certain part of the toy or object (such as turning the wheels of a car) than in playing with the toy as he/she is supposed to?	n	97	102
	%	48.7%	51.3%
Has the child ever had any special hobbies that seem unusual in intensity but are appropriate for his/her age or peer group (trains or dinosaurs)?	n	95	104
	%	47.7%	52.3%
Has the child ever seemed strangely interested in the sight, feel, sound, taste, or smell of things or people?	n	88	111
	%	44.2%	55.8%
Has the child ever shown repetitive and distinctive ways of moving his/her hands or fingers, such as twisting or flapping his/her fingers in front of his/her eyes?	n	77	122
	%	38.7%	61.3%
Has the child ever made any complex movements with his/her whole body, such as spinning or bouncing repeatedly?	n	88	111
	%	44.2%	55.8%
Has the child ever hurt himself/herself intentionally, such as biting his/her arm or hitting his/her head with something?	n	64	135
	%	32.2%	67.8%
Has the child ever had an attachment to a specific thing (other than soft toys or a comfortable blanket) and carries it with him/her wherever he/she goes?	n	77	122
	%	38.7%	61.3%
Does the child have specific or close friends?	n	128	71
	%	64.3%	35.7%
When he/she was four to five years old, did the child talk to you just to chat (instead of to get something)?	n	122	77
	%	61.3%	38.7%

The data collected regarding the participants’ knowledge of the association of screen time exposure with ASD in children are shown in Table 4. The parameters encompassed a wide range of social and communicative behaviors exhibited by children at a young age, such as imitating others, pointing to objects, using gestures for communication, expressing approval or rejection through nodding and shaking the head, maintaining eye contact during interactions, responding to smiles, sharing interests and favorite things, showing empathy toward others, engaging in social games and imaginative play, and participating in cooperative activities with peers. The data showed the diversity in responses among the participants, shedding light on the varying levels of these behaviors observed in children within the specified age group.

**Table 4:** Participants’ knowledge of the association of screen time exposure with autism spectrum disorder in children. Data are presented as numbers and percentages (n = 199).

Parameter	Responses	Yes	No
When he/she was four to five years old, did the child automatically imitate you or other people by doing something you did, such as sweeping the house, gardening, or repairing things?	N	133	66
	%	66.8%	33.2%
When he/she was four to five years old, did the child automatically point to things around him/her so that he/she could see them, not because he/she wanted them?	N	116	83
	%	58.3%	41.7%
When he/she was four to five years old, did the child use gestures other than pointing or pulling your hand to tell you what he/she wanted?	N	95	104
	%	47.7%	52.3%
When he/she was four to five years old, did the child nod his/her head to express approval?	N	122	77
	%	61.3%	38.7%
When he/she was four to five years old, did the child shake his/her head to express rejection?	N	118	81
	%	59.3%	40.7%
When he/she was four to five years old, did the child usually look directly at your face when he/she did something with you or talked to you?	N	133	66
	%	66.8%	33.2%
When he/she was four to five years old, did the child smile back if someone smiled at him/her?	N	141	58
	%	70.9%	29.1%
When he/she was four to five years old, did the child show you things he/she was interested in to attract your attention?	N	132	67
	%	66.3%	33.7%
When he/she was four to five years old, did the child offer you to share his/her favorite things other than food?	N	127	72
	%	63.8%	36.2%
When he/she was four to five years old, did the child seem to want to share his/her enjoyment of something?	N	140	59
	%	70.4%	29.6%
When he/she was four to five years old, did the child try to comfort you if you were sad or hurt?	N	122	77
	%	61.3%	38.7%
When he/she was four to five years old, if the child wanted something or wanted help, did he/she look at you and use gestures with sounds or words to attract your attention?	N	135	64
	%	67.8%	32.2%
When he/she was four to five years old, did the child show the normal range of facial expressions?	N	137	62
	%	68.8%	31.2%
When he/she was four to five years old, did the child spontaneously engage in social games?	N	131	68
	%	65.8%	34.2%
When he/she was four to five years old, did the child play any role in simulation (pretend) games?	N	121	78
	%	60.8%	39.2%
When he/she was 4-5 years old, did the child seem interested in other unfamiliar children who were about the same age as him/her?	N	109	90
	%	54.8%	45.2%
When he/she was four to five years old, did the child respond positively when another child approached him/her?	N	138	61
	%	69.3%	30.7%
When he/she was four to five years old, if you entered a room and started talking to the child without calling him/her by name, did he/she usually look and pay attention to you?	N	141	58
	%	70.9%	29.1%
When he/she was four to five years old, did the child participate in imaginative play with another child in a way that both seemed to understand what the other was acting out?	N	124	75
	%	62.3%	37.7%
When he or she was four to five years old, did the child play cooperatively with others in games that require participation with a group of children, such as tag and football?	N	151	48
	%	75.9%	24.1%

Social communication scores among children enrolled in the study revealed that 48.2% of the sample (96 children) exhibited good social communication skills, while 51.8% (103 children) demonstrated poor social communication abilities. It is noteworthy that the total sample size comprised 199 individuals, indicating a substantial dataset for analysis. The stark contrast between the percentages of good and poor social communication scores suggests a notable disparity within the cohort, warranting further investigation into potential factors influencing these outcomes. Moreover, the findings underscore the importance of addressing social communication skills in children, as they play a pivotal role in their overall development and interactions with peers and society at large.

The relationship between social communication skills and sociodemographic characteristics among enrolled study participants is shown in Table 5. Data showed that social communication in children in Arar City had a statistically significant relationship with the mother’s age at birth (p = 0.050), mother’s education level (p = 0.009), father’s education level (p = 0.049), whether the child was suffering from any chronic or neurological disease (p = 0.008), age since the child was exposed to electronic devices (p = 0.049), and screen time exposure duration (p = 0.040). It also showed a statistically insignificant relationship with the mother’s occupation, father’s occupation, mother’s marital status, family household income, whether there was a degree of consanguinity between the mother and the father, number of family members, child’s age, gender, birth order, and whether the child’s birth was premature. The multivariate logistic regression model showed that chronic or neurological diseases (p = 0.002, odds ratio = 1.5, 95% confidence interval = 1.2-1.8) and screen time exposure (p = 0.03, odds ratio = 1.15, 95% confidence interval = 1.05-1.3) were the main variables affecting the outcomes.

**Table 5:** Relationship between social communication skills and sociodemographic characteristics. Data are represented as numbers and percentages. P-values were estimated using the chi-square test. *: p < 0.05; **: p < 0.001.

		Responses	Social communication skills		Total (N = 199)	P-value
			Good	Poor		
Mother’s age at birth	Less than 20	N	6	16	22	0.050
		%	6.3%	15.5%	11.1%	
	20 to 34	N	77	68	145	
		%	80.2%	66.0%	72.9%	
	35 or above	N	13	19	32	
		%	13.5%	18.4%	16.1%	
Mother’s occupation	Government employee	N	42	46	88	0.229
		%	43.8%	44.7%	44.2%	
	Private sector employee	N	2	5	7	
		%	2.1%	4.9%	3.5%	
	Housewife	N	52	49	101	
		%	54.2%	47.6%	50.8%	
	Others	N	0	3	3	
		%	0.0%	2.9%	1.5%	
Mother’s education level	Primary school	N	3	10	13	0.009**
		%	3.1%	9.7%	6.5%	
	Middle school	N	7	18	25	
		%	7.3%	17.5%	12.6%	
	High school	N	5	8	13	
		%	5.2%	7.8%	6.5%	
	College degree or higher	N	76	57	133	
		%	79.2%	55.3%	66.8%	
	Uneducated	N	5	10	15	
		%	5.2%	9.7%	7.5%	
Father’s occupation	Government employee	N	73	83	156	0.594
		%	76.0%	80.6%	78.4%	
	Private sector employee	N	11	7	18	
		%	11.5%	6.8%	9.0%	
	Retired	N	1	0	1	
		%	1.0%	0.0%	0.5%	
	Freelancer	N	3	5	8	
		%	3.1%	4.9%	4.0%	
	Unemployed	N	8	8	16	
		%	8.3%	7.8%	8.0%	
Father’s education level	Primary school	N	1	5	6	0.049*
		%	1.0%	4.9%	3.0%	
	Middle school	N	5	10	15	
		%	5.2%	9.7%	7.5%	
	High school	N	23	17	40	
		%	24.0%	16.5%	20.1%	
	College degree or higher	N	65	62	127	
		%	67.7%	60.2%	63.8%	
	Uneducated	N	2	9	11	
		%	2.1%	8.7%	5.5%	
Mother’s marital status	Married	N	92	92	184	0.169
		%	95.8%	89.3%	92.5%	
	Divorced	N	2	8	10	
		%	2.1%	7.8%	5.0%	
	Widowed	N	2	3	5	
		%	2.1%	2.9%	2.5%	
Degree of consanguinity between the mother and the father	No	N	41	42	83	0.782
		%	42.7%	40.8%	41.7%	
	Yes	N	55	61	116	
		%	57.3%	59.2%	58.3%	
Family household income in SAR	Less than 5,000	N	5	7	12	0.438
		%	5.2%	6.8%	6.0%	
	5,000 to 10,000	N	41	35	76	
		%	42.7%	34.0%	38.2%	
	More than 10,000	N	50	61	111	
		%	52.1%	59.2%	55.8%	
Number of family members	Less than 4	N	31	22	53	0.208
		%	32.3%	21.4%	26.6%	
	From 4 to 5	N	24	32	56	
		%	25.0%	31.1%	28.1%	
	More than 5	N	41	49	90	
		%	42.7%	47.6%	45.2%	
Child’s age	4 years old	N	44	34	78	0.164
		%	45.8%	33.0%	39.2%	
	5 years old	N	17	20	37	
		%	17.7%	19.4%	18.6%	
	6 years old	N	35	49	84	
		%	36.5%	47.6%	42.2%	
Child’s gender	Female	N	30	22	52	0.113
		%	31.3%	21.4%	26.1%	
	Male	N	66	81	147	
		%	68.8%	78.6%	73.9%	
Child’s birth order	First	N	31	28	59	0.544
		%	32.3%	27.2%	29.6%	
	Second	N	12	15	27	
		%	12.5%	14.6%	13.6%	
	Third	N	13	21	34	
		%	13.5%	20.4%	17.1%	
	Fourth or more	N	40	39	79	
		%	41.7%	37.9%	39.7%	
Premature labor	No	N	83	78	161	0.054
		%	86.5%	75.7%	80.9%	
	Yes	N	13	25	38	
		%	13.5%	24.3%	19.1%	
Does the child suffer from any chronic, neurological, or debilitating disease?	No	N	89	82	171	0.008**
		%	92.7%	79.6%	85.9%	
	Yes	N	7	21	28	
		%	7.3%	20.4%	14.1%	
Age of child since exposed to electronic devices	Less than 2 years	N	21	39	60	0.049*
		%	21.9%	37.9%	30.2%	
	2 to 3 years	N	38	32	70	
		%	39.6%	31.1%	35.2%	
	More than 3 years	N	37	32	69	
		%	38.5%	31.1%	34.7%	
Screen time exposure duration	Less than an hour	N	22	23	45	0.040*
		%	22.9%	22.3%	22.6%	
	1 to 2 hours	N	37	24	61	
		%	38.5%	23.3%	30.7%	
	More than 2 hours	N	37	56	93	
		%	38.5%	54.4%	46.7%	

## Discussion

Children with ASD have impaired social skills due to a brain maturation problem. Children diagnosed with ASD sometimes struggle with typical social interaction and communication [23]. Although a single cause for autism has not been identified, research points to a potential genetic and environmental mix. The Centers for Disease Control and Prevention report that ASD affects one in 44 children and is four times more likely in boys than in girls. Due to social isolation and low self-esteem, which can result in anxiety and despair, people with autism typically have a worse quality of life than people without the condition [24].

Technology will always be a part of our lives owing to advancements in the field. Children are using electronic gadgets more frequently because they are in a developmental stage where they are more susceptible. Studies have shown that exposure to violent media can have detrimental impacts on sleep, physical health (such as obesity and eyesight issues), and behavior (such as aggressive conduct) [25,26]. Nonetheless, there is no denying the advantages of using electronics. Electronic devices, to name a few, have made it easier for kids to socialize during times of frequent lockdowns and limited outdoor activities. Additionally, because kids can now easily access instructional content, it has completely changed the way people learn [27]. As a result, the American Academy of Pediatrics has developed age-appropriate guidelines for kids’ media consumption that weigh the advantages and disadvantages of each medium.

For children ages two to five, the American Association of Pediatrics recommends one hour during the week and three hours on the weekends [10]. When it comes to screen time, it is advised that caregivers spend no more than an hour a day co-watching quality shows with children ages two to five. Over five-year-olds can watch alone as long as they follow stringent guidelines set by the caregiver regarding screen time and program choices. This will help prevent bad outcomes for the child’s behavior, sleep patterns, and other areas of health. Regrettably, present screen usage exceeds the suggested guidelines. Thus, the purpose of this study was to investigate the relationship between early childhood (ages four to six) screen time exposure and the development of ASD in Arar City, KSA.

Concerning the relationship between screen time exposure and ASD, we discovered that there were differences in the length of screen time exposure, with 22.6% of children exposed for less than an hour, 30.7% for one to two hours, and 46.7% for more than two hours. The variety of electronic gadgets that kids were exposed to also differed, with cell phones accounting for the majority (68.3%), followed by iPads (24.1%), personal computers (5.0%), televisions (2.0%), and PlayStations (0.5%). Regarding the age at which children were first exposed to electronic devices, the data showed that 30.2% had been exposed before the age of two, 35.2% between the ages of two and three, and 34.7% after the age of three. However, another study among Chinese preschoolers found that preschoolers were more likely to exhibit autistic tendencies when they were exposed to screens at a young age [28]. Furthermore, a study conducted in Japan revealed that mobile phone use averaged 24 hours a week [29]. In Saudi Arabia, a few studies have been conducted to ascertain the most commonly used devices and the degree of exposure to these devices. The television is the most used gadget, followed by computers, mobile phones, and tablets. Children also use electronics for more than four hours a day [30].

Furthermore, according to a different survey, children use cell phones the most frequently (28.5 ± 27 hours per week on average), followed by tablets (7.5 ± 15 hours) and laptops (3 ± 7.4 hours). According to a study, 35 hours a week was the median time spent using all the gadgets combined [31]. At a higher level, a 2019 study examined how increased screen usage may affect toddlers’ and preschoolers’ development of their white matter. Language, literacy, and cognitive function are all governed by white matter [32].

Numerous studies have discovered a link between excessive screen use and symptoms similar to autism. A 2022 study found a connection between ASD in boys at age three and extended screen time exposure at age one [33]. On the other hand, a European study emphasized the benefits of video gaming for young children, finding that those who played games performed better academically and had higher cognitive functioning [34]. Conversely, Hu et al. [35] discovered that the majority of kids watched television and watched videos for 2.16 (SD = 1.03) hours per day. Furthermore, they reported 1.07 (SD = 0.90) hours of active screen usage, on average, which included using smartphones and computers. This illustrates how a child’s neurodevelopment could be adversely affected by one to two hours of screen time each day, which could lead to ASD. According to Fadzil et al., children with a mean score of 3 on the 20-item parent-report screening tool M-CHAT-R have an increased chance of developing ASD if they spend more than three hours watching screens [36]. Furthermore, Dehiol et al. discovered that individuals with ASD viewed various screens, primarily televisions, for almost four hours per day (p = 0.001) [37]. However, Slobodan et al.’s earlier systematic research [38] also examined the relationship between screen time exposure and the onset of ASD. Their results corroborated our observations.

Our study had some limitations. Being a questionnaire-based study, the results were interpreted according to the parent’s statements regardless of the widely reported recall bias among participants. The health status of the children was noted according to their own statements and physical health characteristics. In addition, the study design could not infer the cause-effect relationship because the data about exposure and outcome were collected simultaneously. Furthermore, limited longitudinal data and no control for the confounding variables could have impacted the observed outcomes.

## Conclusions

This study highlights the significant effect of screen time exposure on the development of ASD. The findings indicate a correlation between increased screen time duration, early age of exposure to electronic devices, and poor social communication skills in children. It is crucial for parents, caregivers, and healthcare professionals to be aware of the potential risks associated with prolonged screen time and to implement guidelines recommended by organizations such as the American Academy of Pediatrics and WHO to ensure healthy development in children. Further research and interventions are needed to better understand and address the impact of screen media use on children’s neurodevelopment and overall well-being.
